# Qiliqiangxin Modulates the Gut Microbiota and NLRP3 Inflammasome to Protect Against Ventricular Remodeling in Heart Failure

**DOI:** 10.3389/fphar.2022.905424

**Published:** 2022-06-02

**Authors:** Yingdong Lu, Mi Xiang, Laiyun Xin, Yang Zhang, Yuling Wang, Zihuan Shen, Li Li, Xiangning Cui

**Affiliations:** ^1^ Department of Cardiovascular, Guang’anmen Hospital, China Academy of Chinese Medical Sciences, Beijing, China; ^2^ First Clinical Medical School, Shandong University of Chinese Medicine, Jinan, China

**Keywords:** gut microbiota, NLRP3 inflammasome, qiliqiangxin, heart failure, ventricular remodeling

## Abstract

**Aims**: Pathological left ventricular (LV) remodeling induced by multiple causes often triggers fatal cardiac dysfunction, heart failure (HF), and even cardiac death. This study is aimed to investigate whether qiliqiangxin (QL) could improve LV remodeling and protect against HF *via* modulating gut microbiota and inhibiting nod-like receptor pyrin domain 3 (NLRP3) inflammasome activation.

**Methods:** Rats were respectively treated with QL (100 mg/kg/day) or valsartan (1.6 mg/kg/day) by oral gavage after transverse aortic constriction or sham surgery for 13 weeks. Cardiac functions and myocardial fibrosis were assessed. In addition, gut microbial composition was assessed by 16S rDNA sequencing. Furthermore, rats’ hearts were harvested for histopathological and molecular analyses including immunohistochemistry, immunofluorescence, terminal-deoxynucleotidyl transferase-mediated 2’-deoxyuridine 5’-triphosphated nick end labeling, and Western blot.

**Key findings:** QL treatment preserved cardiac functions including LV ejection fractions and fractional shortening and markedly improved the LV remodeling. Moreover, HF was related to the gut microbial community reorganization like a reduction in *Lactobacillus*, while QL reversed it. Additionally, the protein expression levels like IL-1β, TNF-α, NF-κB, and NLRP3 were decreased in the QL treatment group compared to the model one.

**Conclusion:** QL ameliorates ventricular remodeling to some extent in rats with HF by modulating the gut microbiota and NLRP3 inflammasome, which indicates the potential therapeutic effects of QL on those who suffer from HF.

## Introduction

As one of the most prevalent cardiac dysfunctions, heart failure (HF) is caused by multiple cardiac diseases like coronary heart disease, hypertension, arrhythmia, and viral myocarditis (Zhong et al., 2021), characterized by high morbidity and mortality (Ren et al., 2021; Sokolski et al., 2022). A significant pathological basis of HF is cardiac remodeling, which is thought to play a key part in the clinical outcomes of heart diseases (Liu et al., 2021) and occurs through various complex mechanisms, leading to changes such as pathological cardiac hypertrophy, interstitial fibrosis, increased degradation of the myocardial extracellular matrix, impaired heart functions, and even HF (Hao et al., 2021; Ni et al., 2022; Ren et al., 2021). Cardiac remodeling in HF is typically characterized by myocardial hypertrophy (Zhong et al., 2021), which is initially an adaptive and compensatory response to maintain the ejection fraction under increased pressure load, while irreversible damage could be induced if pathological overload persists, along with inflammatory responses, vascular dysfunction, increased extracellular matrix deposition, myocardial fibrosis, and all pathologic changes resulting in cardiac dysfunction (Nemska et al., 2021; Tsuda, 2021; Zhong et al., 2021). Although several interventions are currently used in HF treatment, aiming to decrease cardiac afterload, there are no drugs available that target the initial inflammation and myocardial remodeling at the origin of hypertrophy (Nemska et al., 2021). It is, therefore, necessary to thoroughly elucidate the underlying pathogenesis of ventricular remodeling in HF and find new therapeutic targets.

A hypothesis, named “gut hypothesis of heart failure”, has attracted widespread attention recently, in which a decreased cardiac output and elevated systemic congestion were thought to cause intestinal mucosal ischemia and/or edema, increased bacterial translocation, augmented circulating endotoxins, and eventually underlying inflammatory response in HF patients (Tang et al., 2017). Gut microbiota can affect the host *via* various processes, including the interaction of their signaling molecules such as lipopolysaccharides (LPSs) and peptidoglycans with cells on the mucosal surface usually *via* pattern recognition receptors (PRRs) that can affect distal organs directly or indirectly and some other processes (Tang et al., 2017). PRRs are primarily composed of toll-like receptors (TLRs) and nod-like receptor (NLR) inflammasomes, among which nod-like receptor pyrin domain 3 (NLRP3) is the best characterized inflammasome (Herrmann et al., 2021). Accumulating evidence has demonstrated that NLRP3 plays a vital role in LV remodeling in HF. In the transverse aortic constriction (TAC) models, the NLRP3 inflammasome was demonstrated to form in cardiomyocytes where NLRP3 signaling promoted left ventricle maladaptive response to pressure overload (Suetomi et al., 2018) and cause LV dysfunction, hypertrophy, and fibrosis (Dang et al., 2020). The pharmacological inhibition of NLRP3 or NLRP3 knockout reduced myocardial inflammation, fibrosis, myocardial hypertrophy, and cardiac dysfunction (Gan et al., 2018; Willeford et al., 2018; Zhang et al., 2020). Mechanistically, the NLRP3 macromolecules accelerate caspase-1 activation as well as the secretion and accumulation of proinflammatory cytokines like IL-1β and IL-18 afterward (Mauro et al., 2019). Nevertheless, it is uncertain whether blocking NLRP3 can reverse the cardiac remodeling following HF (Zhang et al., 2020).

Qiliqiangxin (QL) is a well-known compound preparation of traditional Chinese medicine with extracts from 11 herbs including astragali radix, ginseng radix et rhizoma, aconiti lateralis radix preparata, salviae miltiorrhizae radix et rhizoma, descurainiae semen lepidii semen, alismatis rhizoma, cinnamomi ramulus, periplocae cortex, carthami flos, polygonati odorati rhizoma, and citri reticulatae pericarpium (Cheng et al., 2020; Fu et al., 2018). QL has been approved by the National Medical Products Administration for the treatment of HF for more than a decade. In addition, a multicenter randomized double-blind study demonstrated the cardioprotective effect of QL in cardiac patients with HF (Li et al., 2013). Moreover, QL has been reported to ameliorate cardiac remodeling through increasing cardiac contractibility and regulating inflammation in the treatment of chronic HF (Xiao et al., 2009). Finally, our previous study has reported that QL can attenuate cardiac remodeling *via* NF-κB signaling pathways in a rat model of myocardial infarction (Han et al., 2018). Most of these studies were focused on PPAR gamma, TGF-beta1/Smads, or cardiac metabolism. Nevertheless, what remains unknown is whether QL prevents hearts from ventricular remodeling through regulating the intestinal function and the NLRP3 inflammasome. In the present study, therefore, we explored the role of QL in ventricular remodeling *via* comparison with valsartan using Sprague–Dawley (SD) rats with the model of TAC. We hypothesized QL’s pharmacological interventions in the microbial community structure and NLRP3-inflammatory corpuscle signaling, which thereby alleviated ventricular remodeling in HF.

## Materials and Methods

### Model Induction of Heart Failure Through Pressure Overload

Male SD rats (220–250 g), purchased from Beijing Vital River Laboratory Animal Technology Co., Ltd. [Animal license number: SCXK (Beijing) 2017–0302], were fed adaptively for 3 days. A model of HF was established surgically through transverse aortic constriction (TAC) operation as has been described (Ruppert et al., 2020), which is one of the most widespread preclinical models of cardiac hypertrophy and HF induced by pressure overload (Boccella et al., 2021). Following anesthesia with sodium pentobarbital (40 mg/kg) and subsequently a left anterolateral thoracotomy, the aortic arch between the brachiocephalic trunk and the left common carotid artery was exposed and then constricted to the size of a 21-gauge needle. The rats in the sham-operation group underwent the same procedure but without aortic constriction. Following the modeling, the surviving rats with HF were randomly divided into three groups, which were the model group (Model, n = 5), QL group (Model + QL, n = 7), and valsartan group (Model + Valsartan, n = 6). There are finally four groups including the sham group (Sham, n = 7) with sham operation. The animals in the four groups were maintained under the same conditions for 13 weeks, which were kept in a relatively constant temperature room (24°C ± 1°C) with a natural day/night cycle light and given ad libitum access to standard chow and water. All the experimental procedures were approved by the Institutional Animal Care and Use Committee of Guang’anmen Hospital, China Academy of Chinese Medical Sciences, in accordance with the regulations on the management and use of experimental animals.

### Interventions

The QL capsule, which was obtained from Shijiazhuang Yiling Pharmaceutical Co., Ltd. (Shijiazhuang, Hebei, China), and valsartan, which was purchased from Beijing Novartis Pharmaceutical Co., Ltd. (Beijing, China) as the positive control, were dissolved separately in sterile water. The rats were respectively treated with QL and valsartan at doses of 100 mg/(kg. d) and 1.6 mg/(kg. d) by oral gavage once a day starting on day 1 following surgery for 13 weeks. The rats in the sham and model groups were given equal volumes of physiological saline solution.

### Echocardiography

Thirteen weeks after the interventions, noninvasive transthoracic echocardiography was performed on rats anesthetized with sodium pentobarbital (40 mg/kg) using an animal-specific Vevo 2100 high-resolution imaging system (Visual Sonics Inc., Toronto, Ontario, Canada). M-mode echocardiograms of the LV short axis were captured at the level of the papillary muscle for determining LV ejection fractions (LVEFs), LV fractional shortening (LVFS), LV end-diastolic anterior wall thickness (LVAWd), LV end-systolic anterior wall thickness (LVAWs), LV end-diastolic posterior wall thickness (LVPWd), and end-systolic posterior wall thickness (LVPWs). These parameters were obtained and averaged from the three cardiac cycles.

### Histopathology, Immunohistochemistry, Immunofluorescence, and TUNEL

After euthanasia, the heart was immediately excised, irrigated clean with cold saline, and transversely dissected into two parts at the maximum transverse diameter. During our experiment, the heart tissues were fixed with 4% paraformaldehyde, embedded in paraffin, and sectioned into 4 μm for hematoxylin and eosin (H&E), Masson, and Sirius Red staining. Image pro plus 6.0 was used to quantify the ratio of fibrosis area to the entire surface.

For assessing the expression of several important proteins through immunohistochemistry, the paraffin slices were put into an autoclave and boiled for 3 min to repair the antigen. After that, the slices were placed at 60°C for 2 h, deparaffinized, hydrated with xylene and ethanol followed by phosphate-buffered saline (PBS), and then incubated with primary antibodies overnight at 4°C: NLRP3 antibody (1:200, R30750, NSJBIO, United States), PYCARD antibody (ASC) (1:200,DF6304, Affinity Biosciences, CHN), cleaved-IL-1β (Asp116) antibody (1:250,AF4006, Affinity Biosciences, CHN),IL1β antibody (1:120, AF5103, Affinity Biosciences, CHN), and TNF-α antibody (1:250, ab66579, Abcam, United States). The slices were afterward incubated with horseradish peroxidase (HRP)-conjugated goat antirabbit immunoglobulin G (IgG) at 37°C for 30 min, followed by staining with a diaminobenzidine (DAB) color kit (ZLI-9017, ZSGB-BIO, and CHN).

For the immunofluorescence staining, the paraffin-embedded colon sections (4 μm) were deparaffinized for citrate-ethylenediaminetetraacetic acid (EDTA) antigen retrieval and incubated with occludin antibodies (1:200 dilution, ab216327, Abcam). Having been placed at 4°C overnight, the colon sections were then washed with PBS and added with a fluorescent secondary antibody. The images were generated by using a confocal laser scanning microscope (Olympus, FV1000) and eventually analyzed by ImageJ.

Apoptosis of cardiomyocytes was finally analyzed through the terminal-deoxynucleotidyl transferase-mediated 2’-deoxyuridine 5’-triphosphated nick-end-labeling (TUNEL) method (Roche, 11684795910).

### Measurement of Gut Microbiota

Gut microbial composition was assessed through 16S rDNA gene sequencing. The rat feces from the cecum were collected using sterile cryotubes and then stored at -80°C until processing, and the DNA sample was extracted using a Power Fecal DNA Isolation Kit (Qiagen-Mobio, German). The V3-V4 variable regions of the 16S rDNA were amplified by PCR; then, a library was constructed. The PCR products were sequenced using an Illumina MiSeq Sequencer (SeqMatic, United States). The raw data were filtered to obtain clean reads, and paired-end reads with the overlap were merged into the tags that were clustered to operational taxonomic units (OTUs) constituting sequences with ≥97% similarity. The Ribosomal Database Project was used to make taxonomic annotation. The complexity of species diversity was analyzed through Chao1 richness, Simpson index, and Shannon index, and the relative abundance of each OTU in each sample was obtained according to the OTU abundance information.

### Western Blot

A total of 20 μg of the cardiac protein extract was separated by 10% or 12% TGX stain-free™ fastcast™ acrylamide kit (1610183/1610185, Bio-Rad, United States) and then transferred to the trans-blot turbo mini 0.2 µm PVDF transfer packs (1704156, Bio-Rad, United States). After blocking with 5% nonfat dry milk (9999S, Cell Signaling, United States) at room temperature for 2 h, we incubated the PVDF membrane with primary antibodies at 4°C overnight: anti-NF-κB p105/p50 antibody (1: 5000, ab32360, Abcam, United Kingdom), NLRP3 antibody/NALP3 (1:500, R30750, NSJBIO, United States), PYCARD antibody (ASC) (1:1000, DF6304, Affinity Biosciences, CHN), cleaved-IL-1β (Asp116) antibody (1:1000, AF4006, Affinity Biosciences, CHN), IL1β antibody (1:1000, AF5103, Affinity Biosciences, CHN), TNF-α antibody (1:1000, ab66579, Abcam, United States), and caspase-1 antibody (D-3) (1:500, sc-392736, Santa Cruz, United States). The PVDF membranes were afterward incubated with the HRP-conjugated goat antirabbit IgG (H&L) (1: 10000, bs-40295G-HRP, Bioss, CHN) or HRP-conjugated goat antimouse IgG (H&L) (1: 1000, A0216, Beyotime, CHN) at room temperature for 1 h. The PVDF membranes were eventually visualized using Gel Doc™ XR + System (Bio-Rad, United States) and Image Lab Software (Bio-Rad, United States) with the ECL substrate (1705062, Bio-Rad, United States), followed by quantification using ImageJ (NIH, United States), and the results expressed as density values were normalized to GAPDH or tubulin.

### Enzyme-Linked Immunosorbent Assay

Concentrations of TNF-α and IL-1β in serum were determined, respectively, using a QuantiCyto^®^ Rat TNF-α ELISA kit (ERC102a.48, NeoBioscience,CHN) and QuantiCyto^®^ Rat IL-1β ELISA kit (ERC007.48,NeoBioscience,CHN). The standard substance at known concentrations and serum were pipetted into the wells of microplate strips, incubated for 90 min at 37°C, away from light. Next, the biotinylated antibodies were incubated for 60 min with TNF-α and the IL-1β antigen, respectively. Then, the bound enzyme and chromogenic substrate were added successively. Finally, The OD_450_ values were detected, which corresponded to the TNF-α and IL-1β concentrations, and the ultimate data were expressed as pg/ml of protein.

### Statistical Analysis

GraphPad Prism software (version 8.0) was used for statistical analysis and drawing of the data, with assays independently repeated at least three times. The values for all the measurements were displayed as the mean ± standard deviation $\left(\bar{x}\pm s\right)$ . Comparisons between multiple groups were performed through repeated one-way analysis of variance (ANOVA), with *p* < 0.05 considered statistically significant.

## Results

### QL Improved Cardiac Functions in Heart Failure

**FIGURE 1:**
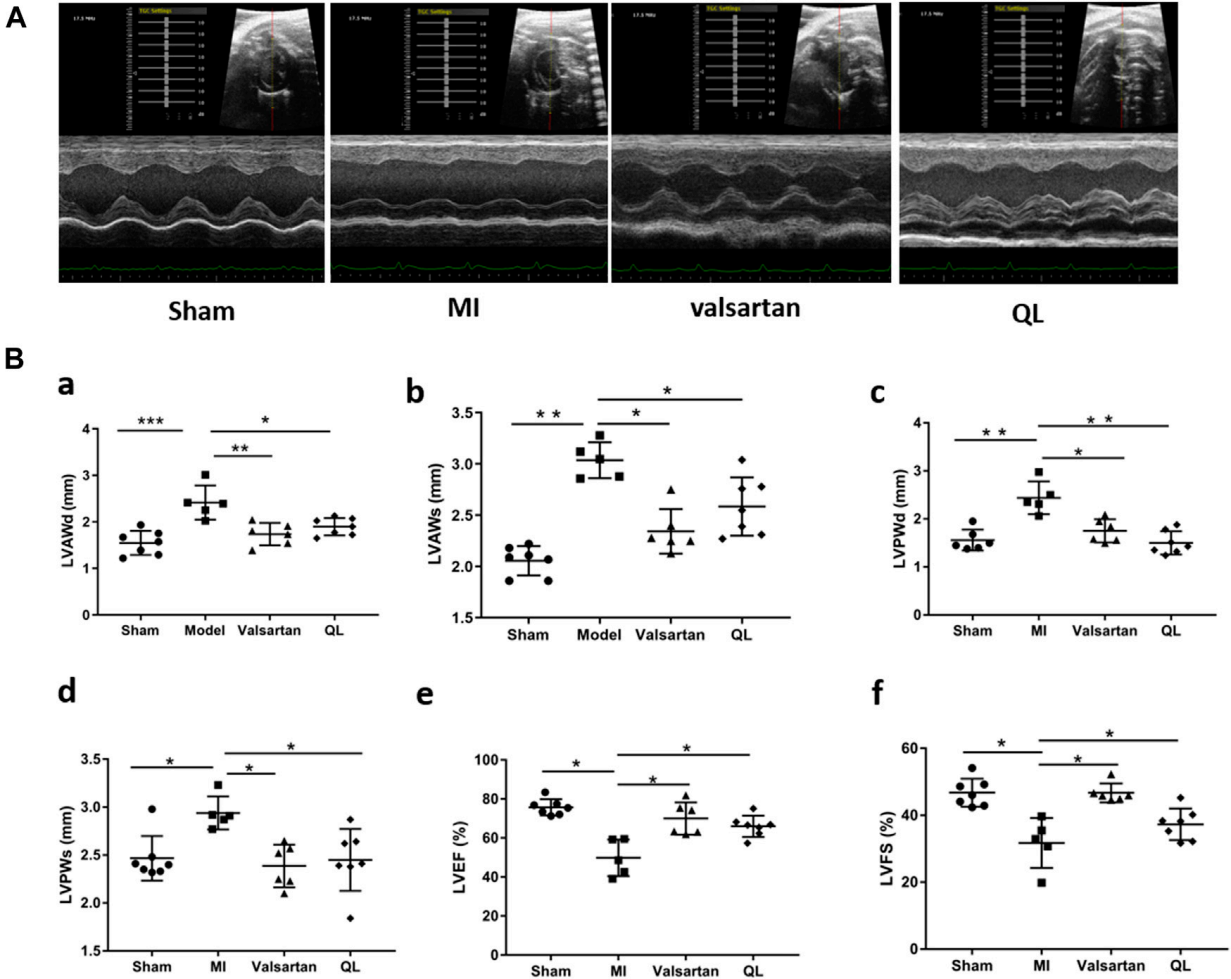
QL protected cardiac functions in HF. **(A)** Representative echocardiograms of rats in four groups. **(B)** Comparison of echocardiography parameters in the four groups. LVAWd: LV end-diastolic anterior wall thicknesses, LVAWs: LV end-systolic anterior wall thicknesses, LVPWd: LV end-diastolic posterior wall thicknesses, LVPWs: LV end-systolic posterior wall thicknesses, LVEF: LV ejection fraction, and LVFS: LV fractional shortening. The data are the mean ± standard deviation $\left(\bar{x}\pm s\right)$ (n = 5, 6, or 7 per group), *p < 0.05, **p < 0.01, and ***p < 0.001.

We first used echocardiography to determine the phenotype establishment of TAC-induced HF and the effects of QL on the cardiac functions. Representative two-dimensional echocardiograms and comparison of various parameters in all groups are illustrated in Figure 1. Significant remodeling manifestations appeared in the model rats 13 weeks following TAC, evidenced by augmentation in LVAWd, LVAWs, LVPWd, and LVPWs (Figure 1B(a–d)). In addition, the cardiac function of the model group was worse than that of the sham group with the reduction in LVEF and LVFS (Figure 1B(e,f)). However, all these parameters were improved by the application of QL versus model group, and no significant difference could be detected between the sham group and QL group.

### QL Reversed LV Remodeling and Cardiomyocytes Apoptosis in Heart Failure

**FIGURE 2:**
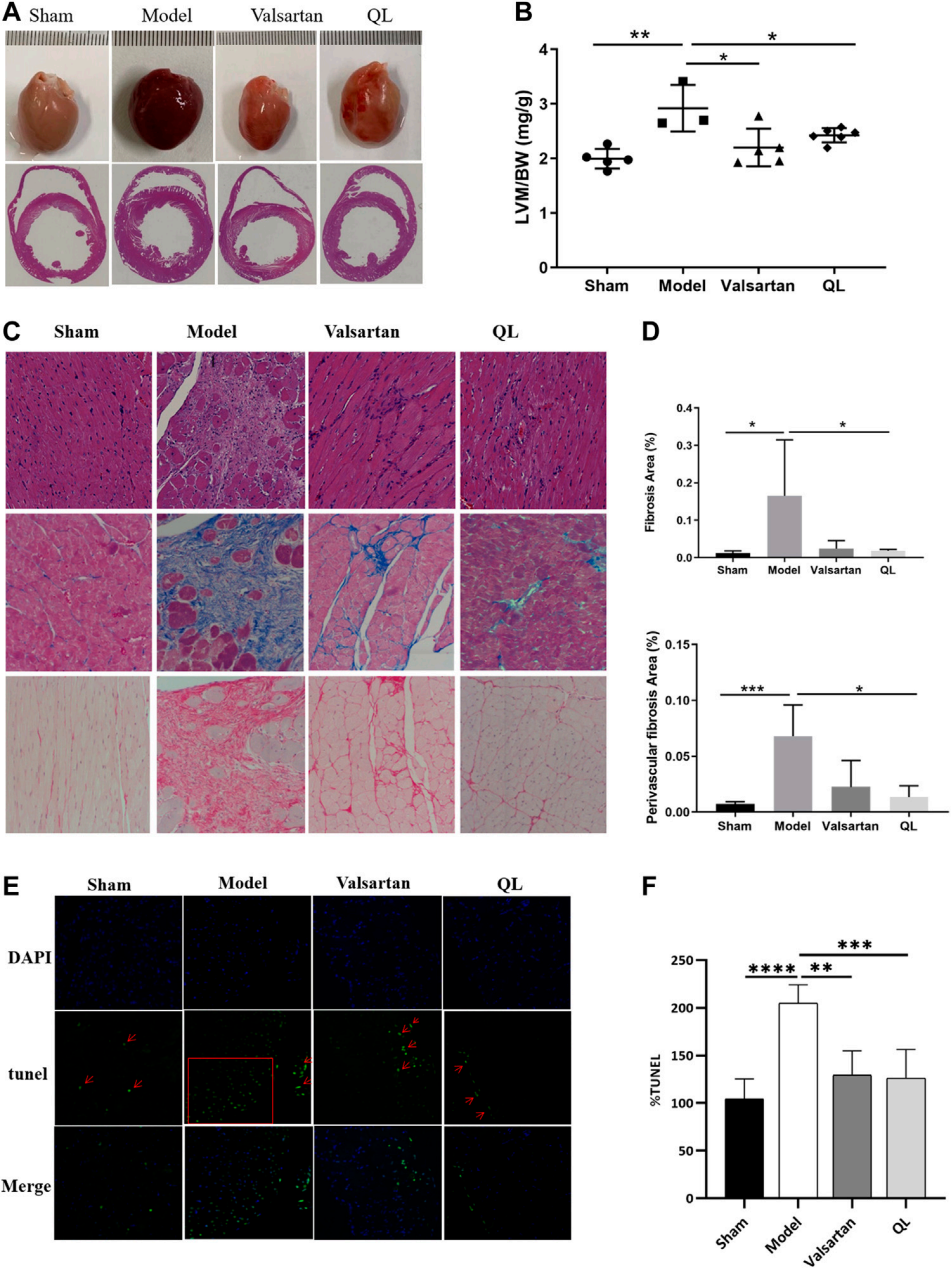
QL reversed ventricular remodeling and alleviate cardiomyocyte apoptosis of rats with HF. **(A)** Appearance and transverse section of the heart. **(B)** Left ventricular mass to total body weight. **(C)** Histopathologic changes of the cardiac tissue with H&E staining and myocardial fibrosis observed with Masson staining and Sirius Red staining (Scale bar = 100 µm). **(D)** Quantification of heart fibrosis area. **(E)** TUNEL staining for cell apoptosis rate. **(F)** Relative apoptosis rate of the myocardial cells. The data are the mean ± standard deviation $\left(\bar{x}\pm s\right)$ (n = 3,4,5, or 6 per group) *p < 0.05, **p < 0.01, ***p < 0.001, ****p < 0.0001.

The results observed through transverse section area of hearts and further quantified by the left ventricular mass to total body weight (LVM/BW) showed slight cardiac hypertrophy in the HF rats and improved conditions by both QL and valsartan (Figure 2A,B). Through the histological analysis and quantification of heart fibrosis area in each group, the irregularly arranged cardiac myocytes, inflammatory infiltration, fibroblast proliferation, fibrous scar formation, and enlarged fibrosis area were observed in the heart of the model rats with HF. The histopathological changes above mentioned were ameliorated by QL and valsartan to varying degrees maintaining the integrity of myocardial cells, and QL appears to display a more significant improvement compared with valsartan (Figure 2C,D). Moreover, as shown in Figure 2E,F for TUNEL staining of the cardiac tissue, the rate of cardiomyocyte apoptosis in the model group increased, while it decreased in both the QL group and valsartan group, especially after QL treatment.

### QL Alters the Composition of Gut Microbiota

**FIGURE 3:**
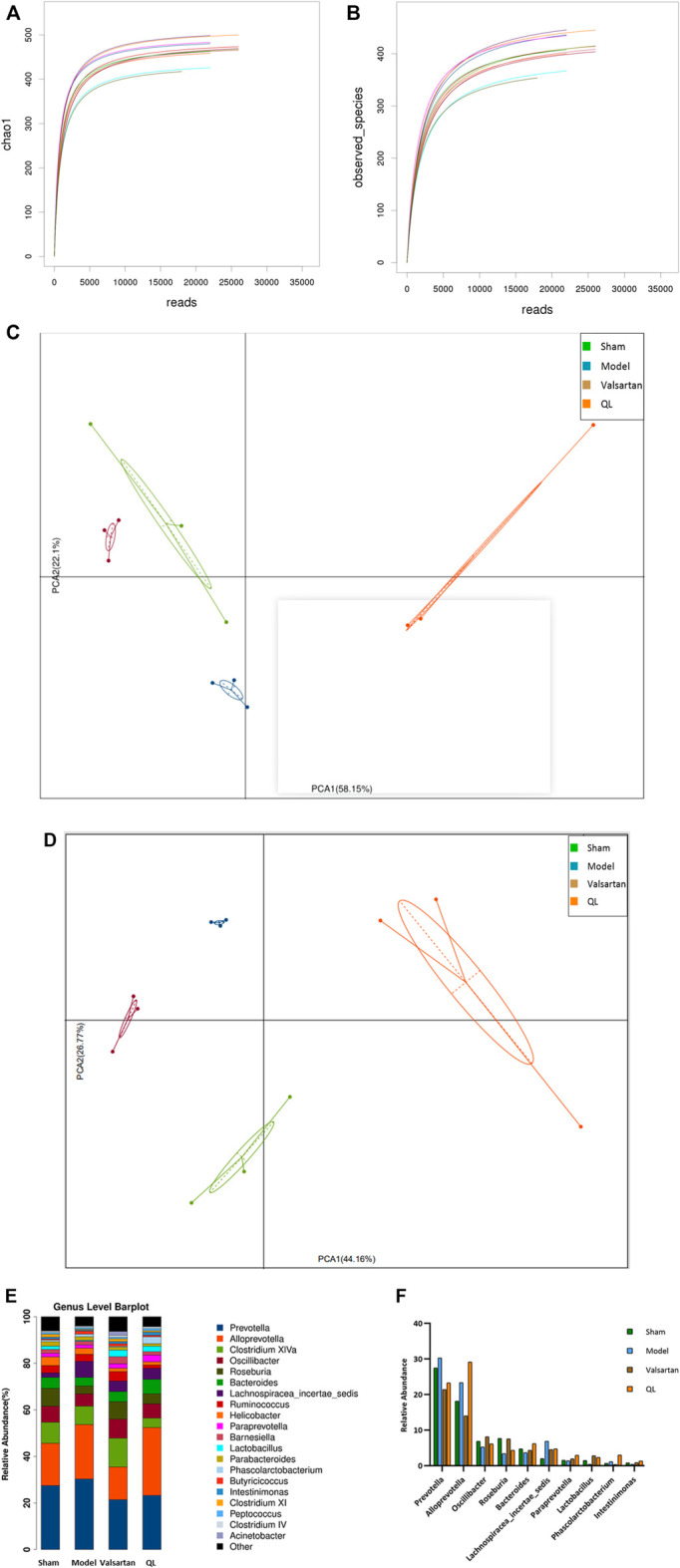
QL regulates the composition of gut microbiota. **(A,B)** Dilution curves of species richness alpha diversity in samples. **(C,D)** PCA results of different microbiota, respectively, in all levels and the genus level. **(E,F)** Relative abundance of gut microbiota at the genus level. The data are the mean ± standard deviation $\left(\bar{x}\pm s\right)$ (n = 3 per group).

When analyzing gut microbiota composition, the operational taxonomic units (OTUs) were obtained by clustering with 97% similarity, and each OTU was considered as a species. As shown in Figure 3A,B, the alpha diversity reflects the species diversity within a single sample, including the observed species index and Chao 1 index, in which each curve represents a sample. The curves of each sample tended to be plateaued as the sequencing depth increased, meaning that the amount of sequencing data is reasonable and the entire microbial community was captured. The principal component analysis (PCA) results of different species in either all the levels or the genus level implied that the flora composition of the four groups can be well distinguished and the differences between each group were well identified (Figure 3C, D). Based on the species annotation results, the top 20 microbiota in abundance at the genus level were selected for each group to generate a column accumulation diagram of relative abundance (Figure 3E). Focused on different expressions among the four groups, our results show differences in the abundance of some microbiota in the HF rats with an increase in Prevotella, Alloprevotella, and Lachnospiracea_incertae_sedis, but these three genera were reduced after valsartan intervention and Prevotella and Lachnospiracea_incertae_sedis in the QL group express lower as well. Some other microbiota in the model group, nevertheless, demonstrated decreased expression including Oscillibacter, Roseburia, *Bacteroides*, and *Lactobacillus*, and those of the valsartan group and QL group were conversely increased to some extent. In addition, through QL intervention, Paraprevotella, Phascolarctobacterium, and Intestinimonas increased significantly compared with the other three groups. The relative abundances of significantly different microbiota at the genus level are exhibited in Figure 3F.

### QL Improves the Gut Barrier

**FIGURE 4:**
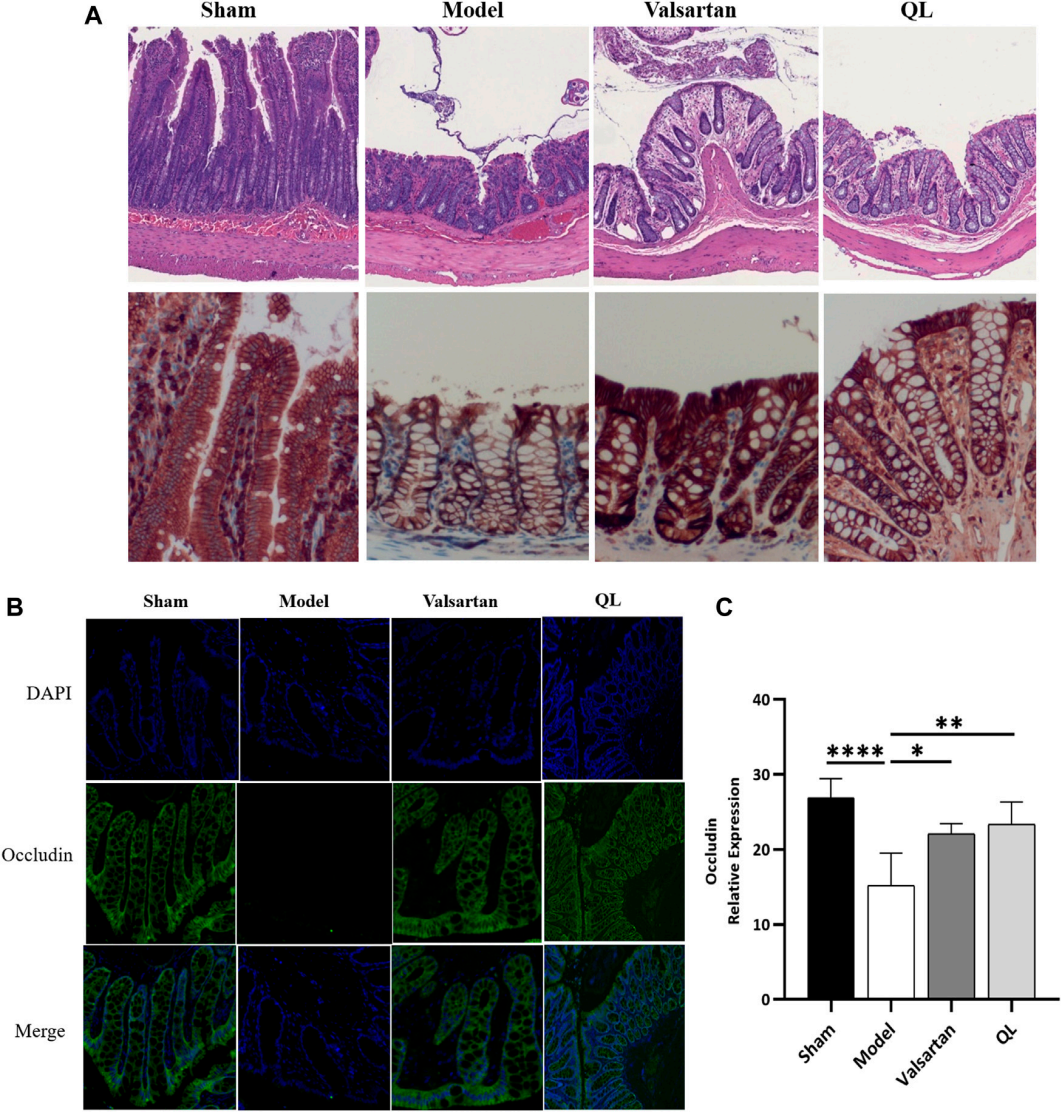
QL ameliorates the pathological damage of the intestinal barrier in HF. **(A)** Pathological changes of the colonic mucosa under a light microscope with H&E staining (scale bar = 300 µm). **(B,C)** Expression of occludin detected with immunofluorescence staining. The data are the mean ± standard deviation $\left(\bar{x}\pm s\right)$ (n = 5 or 6 per group); *p < 0.05, **p < 0.01, and ****p < 0.0001.

In order to confirm the protection of QL on the gut barrier, we evaluated the intestinal injury in the colon of rats in four groups. As shown in Figure 4A, the HF animals were observed with the edematous, severed, or denuded intestinal villi, along with the significantly increased gap between the epithelial cells, which were, however, ameliorated after receiving QL and valsartan treatment. Furthermore, the intestinal epithelial tight junction protein occludin was used as an indicator to evaluate the function of the intestinal barrier, whose expression was disrupted following HF but restored by QL and valsartan nearly to the normal levels (Figure 4B,C).

### QL Inhibits the Expression of NF-κB-Related Signaling Molecules

**FIGURE 5:**
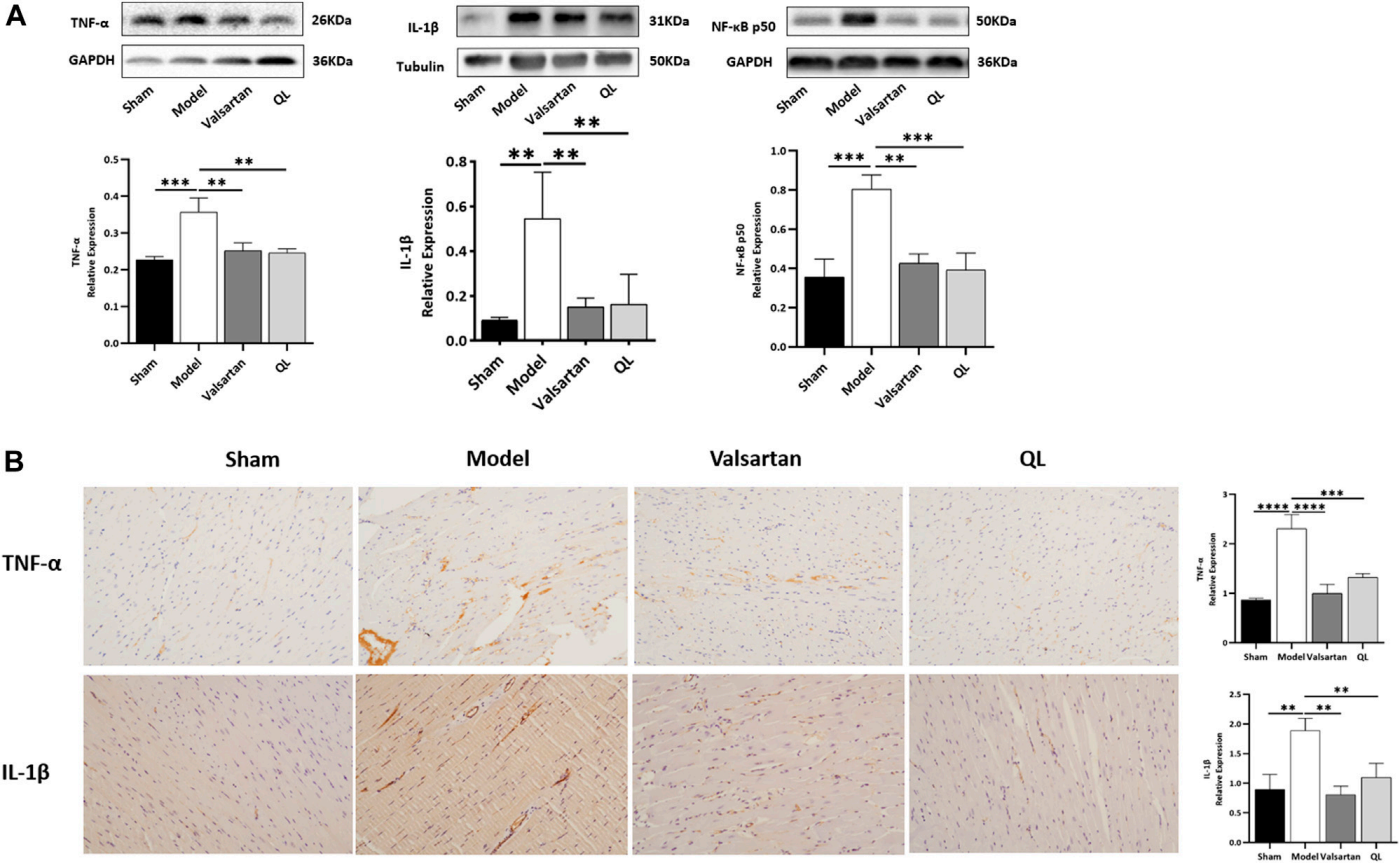
QL suppresses IL-1β, NF-κB, and TNF-α expression. **(A)** Western blot analysis of IL-1β, TNF-α, and NF-κB p50 expression in the cardiac tissue. **(B)** Relative expression of TNF-α and IL-1β in the heart detected through immunohistochemical analyses. GAPDH or tubulin was detected as the loading control. IL-1β: interleukin 1β, NF-κB: nuclear factor kappa B, and TNF-α: tumor necrosis factor-α. The data are the mean ± standard deviation $\left(\bar{x}\pm s\right)$ (n = 3 or 4 per group); **p < 0.01, ***p < 0.001, and ****p < 0.0001.

Subsequently, the levels of IL-1β, NF-κB, and TNF-α in the heart tissue were respectively detected by Western blot and immunohistochemical analysis to determine how disrupted gut microbiota and the damaged gut barrier affect the heart. The results showed that the expressions of IL-1β, NF-κB, and TNF-α were increased in the cardiac tissue of modeling rats relative to those of the sham one but decreased following QL and valsartan treatment (Figure 5A,B). Consistent with these results, the serum levels of proinflammatory cytokines IL-1β and TNF-α were rapidly and persistently enhanced after TAC surgery but decreased after the interventions of QL and valsartan (Supplementary Figure S1A-B).

### QL Inhibited Inflammasomes in Heart Failure

To further verify the pathologic changes shown through inflammasomes, the relative tissue protein expression levels of NLRP3 and its associated proteins in the cardiac tissue were detected through Western blot and immunohistochemistry. The results revealed significantly higher NLRP3 generation in the model group compared with the healthy control group, while treatment of valsartan and QL blocked these effects. Moreover, the protein expressions of ASC, caspase-1, and cleaved-IL-1β were upregulated in the model group but downregulated *via* valsartan and QL intervention, especially the latter one. These results inspired us to conclude that NLRP3 and its associated signaling pathways might be the targets of QL in the pathological process of ventricular remodeling.

## Disscussion

As previously mentioned, cardiac remodeling, caused or exacerbated by varying pathological changes, will eventually result in serious consequences, for which there are no effective drugs (Hao et al., 2021; Ni et al., 2022; Ren et al., 2021). New therapeutic strategies are needed to be introduced clinically for protecting against pressure overload-induced cardiac injury (Li X. et al., 2021) or targeting adverse post-MI left ventricle remodeling (Germano et al., 2020). Researchers highlight the potential of intestinal microbiota and NLRP3 inflammasomes to impact ventricular remodeling, which hold promise as effective targets for improving cardiac functions in HF (Boccella et al., 2021; Zhao et al., 2021). The beneficial effects of QL on cardiac hypertrophy, ventricular remodeling, and HF through multiple pathways have been widely reported (Gao et al., 2018; Lu et al., 2020; Wang et al., 2017). Valsartan, an angiotensin II (ANG II) receptor blocker, exerts positive effects in improving chronic inflammation, promoting scar repair, maintaining cardiomyocyte integrity, alleviating ventricular remodeling, and thus preserving cardiac functions (Shi et al., 2020). Further studies indicated that valsartan administration could affect the intestinal microbiota composition and SCFA generation and blunted the LPS-induced elevations of inflammatory cytokines (Iwashita et al., 2013; Qi et al., 2021). In the present study, therefore, with valsartan as the control intervention, we verified the effects of QL on ventricular remodeling of rats with HF. We found the protective effects of QL on the LV remodeling with the regulation of gut microbiota. Furthermore, we revealed that QL intervention could block the inflammasome formation and inflammatory cytokine release in the cardiomyocytes, which were consistent with those from the previous studies (Zhang et al., 2019; Zou et al., 2012).

Ventricular remodeling involves the alteration of the ventricular structure, with the geometry changes including conversation in wall thickness and cavity diameter, along with a progression from elliptical to spherical configuration (Tomoaia et al., 2019). In addition, it might result in the deterioration of cardiac functions like the declined systolic function (Zhang XX. et al., 2021). The reduced parameters like LVAWd, LVAWs, LVPWd, and LVPWs, along with the slightly improved LVEF and LVFS, showed that pressure overload-induced deterioration of the cardiac structure and functions was partially reversed by QL, suggesting the salutary role of QL against remodeling in HF. Mechanistically, it was thought that ventricular remodeling is primarily attributed to cardiomyocyte reduction and poor development of surviving cardiomyocytes and the extracellular matrix (Zhang XX. et al., 2021). When chronic LV pressure overloads, the cardiomyocytes become hypertrophied and alter their cellular properties, whereas cardiac fibroblasts convert into an activated state, proliferate, and/or increase extracellular matrix deposition (Li X. et al., 2021). These pathological changes ultimately lead to the HF development and poor prognosis (Li X. et al., 2021). The results of histological analysis and fibrosis area quantification additionally revealed the ameliorated histopathological changes by QL to a certain extent.

A growing body of evidence derived from the epidemiological and animal model studies uncovers that altered gut microbiota and gut functions are closely related to the development of adverse cardiac remodeling and HF (Boccella et al., 2021; Carrillo-Salinas et al., 2020). In chronic heart failure patients, the wall thickness and permeability of the intestine increase, the bacterial populations adhering to the intestinal mucosa release, and bacteria or their toxins transfer from the intestine to the blood directly, which associate with systemic inflammation (Wang et al., 2021). Another research, as previously described, demonstrated that decreased cardiac output and elevated systemic congestion may cause intestinal mucosal ischemia and/or edema, increased bacterial translocation, increased circulating endotoxins, and eventually underlying inflammatory response in the HF patients (Tang et al., 2017). In the model of TAC, it was proposed that gut hypoperfusion induced by TAC may lead to intestinal microbiome dysregulation and increased gut permeability, which in turn affect systemic inflammation and contribute to cardiac dysfunction (Boccella et al., 2021). Persistent inflammation of the heart is considered as a sign of hypertrophy of myocardial hypertrophy (Zhong et al., 2021). Consequently, new challenge lying ahead is that specific microbiota, metabolites, or other microbiota-targeted/driven interventions need to be further explored in the human clinical intervention studies. The results shown in Figure 3C,D with the differences in the species composition and abundance in the four groups indicate that QL and valsartan may regulate the intestinal components. From the column accumulation diagram of relative abundance (Figure 3E,F), our results show underexpression of Prevotella and Lachnospiracea_incertae_sedis in the QL group compared with the model group, while some other microbiota including Oscillibacter, Roseburia, *Bacteroides*, *Lactobacillus*, Paraprevotella, Phascolarctobacterium, and Intestinimonas were upregulated *via* QL intervention. The relative abundance changes of Prevotella were reported to injure the colonic epithelia, resulting in inflammation and thus inflammatory cytokine generation like IL-6, TNF-a, and IL-8 (Xia et al., 2021). It is also related to TMAO production (Sardu et al., 2021). Oscillibacter (Chen et al., 2020; Dessì et al., 2021), Roseburia (Cornuault et al., 2020), *Bacteroides* (Yuan et al., 2021), Paraprevotella (Fei et al., 2019; Zhang X. et al., 2021), Phascolarctobacterium (Pan et al., 2021), and Intestinimonas (Afouda et al., 2019; Luo et al., 2021) exert promoting effects in the SCFA generation or associate positively with its content. Oscillibacter, furthermore, exhibited the potential of anti-inflammation and an intestinal defense barrier (Chen et al., 2021). A study in mice revealed the alleviating effect of *Bacteroides* on the LPS-induced inflammation like restoration of LPS-induced TNF-α and IL-10 generation, then reducing the intestinal microbiota disorders, and maintaining the intestinal epithelium integrity and plasma LPS concentration (Tan et al., 2019). *Lactobacillus*, a well-known probiotic, is essential in maintaining intestinal barrier functions and integrity (Li L. et al., 2021), protecting hearts after MI (Li et al., 2021b; Tang et al., 2019), reducing cardiac hypertrophy and HF following MI, and improving LVEF and LVFS (Li L. et al., 2021). Additionally, we have shown the impaired gut barrier in the model group, including the damaged intestinal mucosa and a lower expression of tight junction protein occludin, while it is improved with varying degrees after treatment (Figure 4). Taken together, our findings suggest that QL has partly protective effects on the gut dysbiosis and gut barrier functions, thus protecting the heart from ventricular remodeling and HF.

**FIGURE 6:**
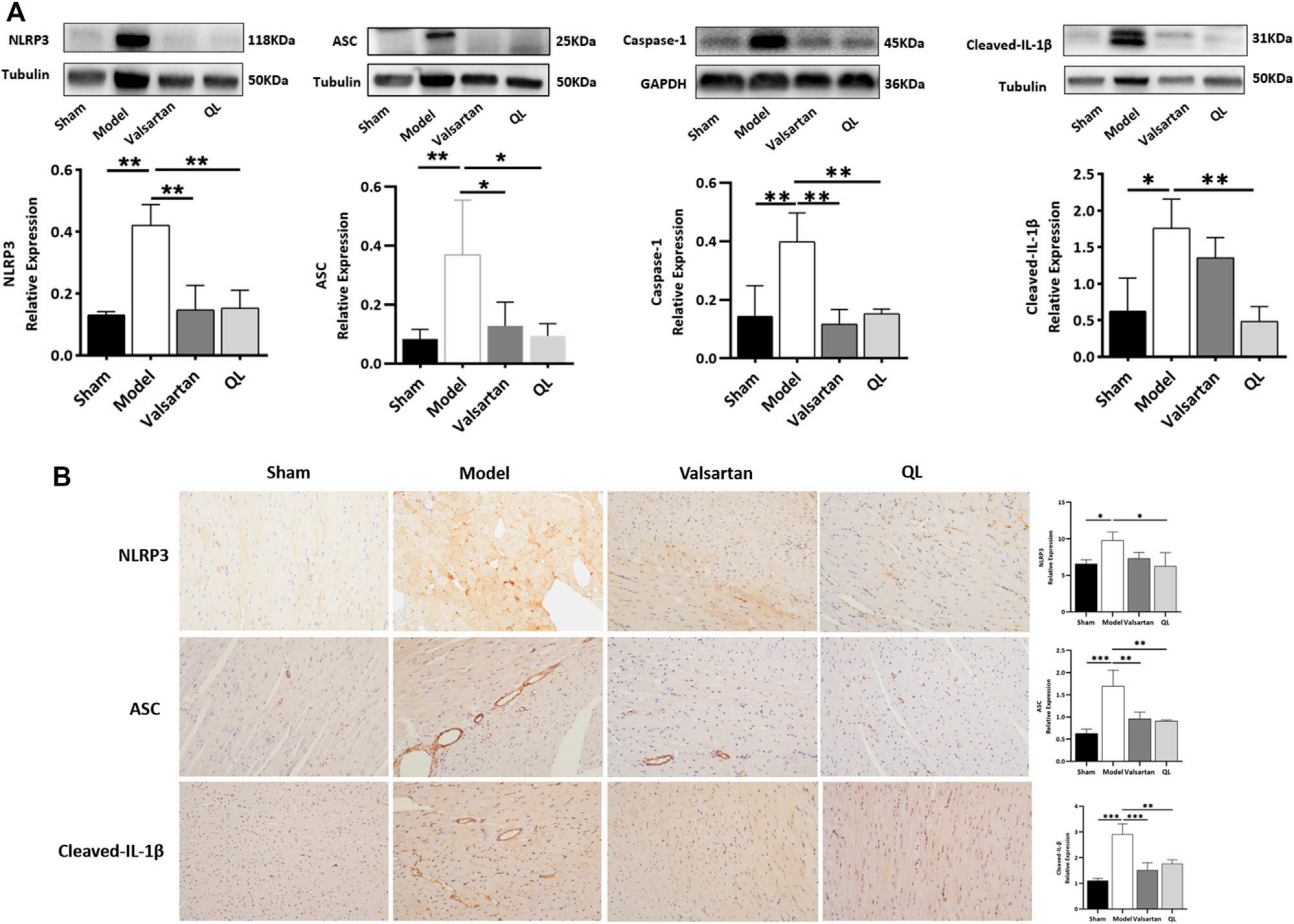
QL inhibits inflammasome generation in HF **(A)** Western blot analysis of NLRP3, ASC, caspase-1, and cleaved-IL-1β protein expression in the cardiac tissue. **(B)** Immunochemical staining of NLRP3, ASC, and cleaved-IL-1β. Tubulin or GAPDH was detected as the loading control. NLRP3: nod-like receptor pyrin domain 3, ASC: apoptosis-associated speck-like protein, and IL-1β: interleukin 1β. The data are the mean ± standard deviation $\left(\bar{x}\pm s\right)$ (n = 3 or 4 per group) *p < 0.05, **p < 0.01, and ***p < 0.001.

Recently, a lot of literature studies have implied that an imbalanced composition and function of the gut mirobiome, known as dysbiosis, increases the risk of incident adverse cardiovascular events, including HF (Go et al., 2013). The current “gut hypothesis of heart failure” first proposed by Tang et al. implies that a decreased cardiac output and adaptive redistribution of systemic circulation lead to the intestinal hypoperfusion, intestinal villi ischemia, bowel wall oedema, and impaired barrier functions (Tang et al., 2019). This disruption in the intestinal barrier function in turn leads to the increase of gut permeability and circulating endotoxins (LPS), augment inflammatory-related responses, and escalated HF (Pasini et al., 2016; Sandek et al., 2014). Gut-derived LPS could trigger inflammatory responses *via* TLRs, which mediates the activation of NF-κB (Chen et al., 2016). NF-κB, a master regulator of the inflammatory gene expression, is activated in various heart diseases like congestive HF and myocardial hypertrophy (Lorenzo et al., 2011). Growing reports showed that the NF-κB pathway plays a critical role in regulating the NLRP3 inflammasome (Afonina et al., 2017; Barroso et al., 2016; Lorenzo et al., 2011). A research revealed that alarmins released on necrotic cell death after AMI, such as TNF and IL-1β, can also act as beginning proinflammatory paracrine responses (Cohen et al., 2015; Dinarello et al., 2013; Franchi et al., 2009), which additionally activate NF-κB and ultimately lead to the transcriptional upregulation of the NLRP3 inflammasome. The NLRP3 inflammasome can in turn lead to cardiac fibrosis and hypertrophy *via* generating inflammatory cytokines such as IL-1β, IL-18, TNF-α, and TGF-β (Yamaguchi et al., 2022). The inflammasome is involved in cardiac fibrosis development in HF, which can be activated under stress and formed with NLRP3 and procaspase-1 by the adaptor protein ASC. It was reported that the NLRP3 inflammasome exerts inflammatory effects through regulating proinflammatory cytokine release, including IL-1β and IL-18, which aggravate the cardiomyocyte dysfunction and cause ventricular remodeling and HF (Dang et al., 2020). The NLRP3 inflammasome, according to another report, could be upregulated and activated in the cardiac hypertrophy models, which aggravates cardiac fibrosis through activating the TGF-β/Smad pathway and promotes cardiac hypertrophy through activating the MAPK pathway (Pan et al., 2019). Altogether, the activation of signaling pathways related to NF-κB and NLRP3 is undoubtedly a key process involved in cardiac remodeling. QL has been shown to inhibit the NF-κB signaling pathway activation in the previous studies (Han et al., 2018), which is consistent with our findings (Figure 5). Furthermore, as shown in Figure 6, we first demonstrated that QL treatment in HF could inhibit the expression of NLRP3, ASC, caspase-1, and cleaved IL-1β, which are NLRP3 inflammasome key proteins (Zhang et al., 2022).

## Conclusion

**FIGURE 7:**
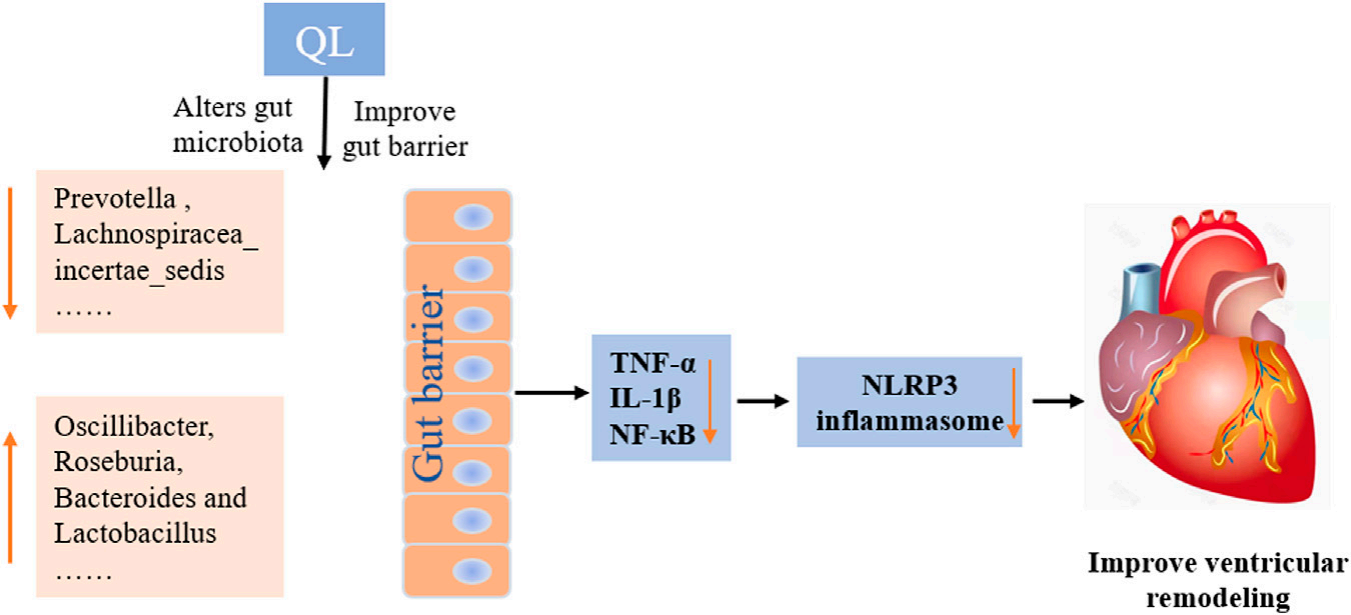
Mechanisms of QL in ventricular remodeling of the transverse aortic constriction-induced HF model. QL intervention could ameliorate the gut dysbiosis, improve the gut barrier, and inhibit gut-derived inflammatory cytokines entering the circulatory system. The heart-protective effect of QL might associate with the NF-κB relative pathway inhibition and the NLRP3 inflammasome suppression in the cardiac tissue.

In conclusion, our current findings indicate that QL treatment is capable of ameliorating ventricular remodeling by inhibiting myocardial fibrosis and apoptosis and improves cardiac functions in the rat models with TAC. This study extends the understanding of the protective effect of QL, indicating that it alters the composition of gut microbiota and intestinal barrier functions and exerts potent anti-inflammatory effects by inhibiting the NLRP3 inflammasome activation (Figure 7). QL might, therefore, be a promising drug for ventricular remodeling and even HF.

## Data Availability

The datasets presented in this study can be found in online repositories. The names of the repository/repositories and accession number(s) can be found below: https://www.ebi.ac.uk/metabolights/, MTBLS4675.
