# Supplementary material for: Qiliqiangxin Modulates the Gut Microbiota and NLRP3 Inflammasome to Protect Against Ventricular Remodeling in Heart Failure
Source: Front Pharmacol. 2022 Jun 2;13:905424. doi: 10.3389/fphar.2022.905424 (PMC9201726; doi:10.3389/fphar.2022.905424)
Supplement: Supplementary file 1 [file Presentation1.pdf]

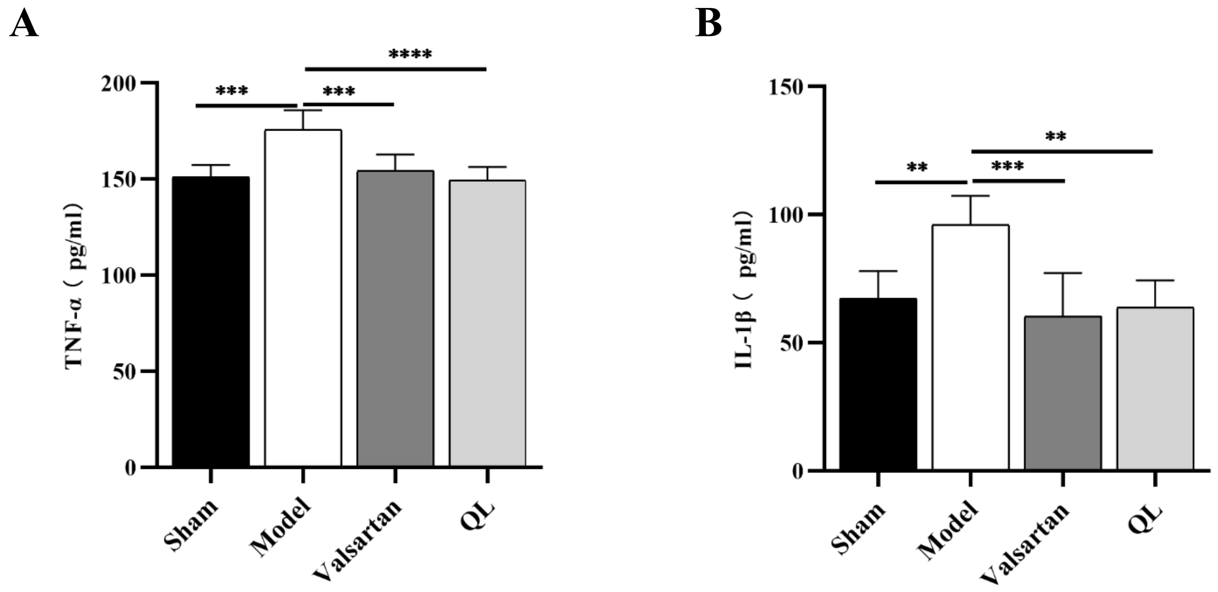


**Fig.S1** | QL suppresses TNF-α and IL-1β in the circulatory system. (A，B) Enzyme-linked immunosorbent assay respectively for detecting TNF-α and IL-1β expression in serum. TNF-α：tumor necrosis factor-α; IL-1β：interleukin 1β. Data are mean ± standard deviation（$\bar{x}$±s） (n = 6 or 7 per group); **P < 0.01, ***P < 0.001, ****P < 0.0001.
